# Preparation and analysis of photochromic behavior of carboxymethyl chitin derivatives containing spiropyran moieties

**DOI:** 10.1080/15685551.2020.1796362

**Published:** 2020-07-26

**Authors:** Bin-Bin Sun, Bing-Hua Yao, Zheng-Sheng Fu, Yang-Qing He

**Affiliations:** a College of Materials Science and Engineering, Xi’an University of Technology, Xi’an, China; b Department of Chemical Engineering, Shaanxi Vocational and Technical College of Defense Industry, Xi’an, China; c College of Chemistry and Chemical Engineering, Northwest Normal University, Lanzhou, China

**Keywords:** Carboxymethyl chitin, spiropyran, graft copolymer, water solubility, negative photochromism, electrostatic attraction

## Abstract

1ʹ-(2-Acryloxyethyl)-3,3ʹ-dimethyl-6-nitrospiro[2 *H*-1-benzopyran-2,2ʹ-indoline] (SPA) was synthesized and grafted onto a water-soluble carboxymethyl chitin (CMCH) macromolecule to prepare a photochromic copolymer (CMCH-g-SPA). The structure of CMCH-g-SPA was characterized by Fourier-transform infrared (FT-IR) spectroscopy, thermogravimetric (TG) analysis, X-ray diffraction (XRD) analysis, water-solubility evaluation, and UV-vis spectroscopy. XRD patterns of CMCH-g-SPA revealed that grafting copolymerization disrupts the CMCH semicrystalline structure, thus improving water solubility. UV-vis spectroscopy results supported the negative photochromic behavior of the merocyanine (MC) form of CMCH-g-SPA (CMCH-g-MCA) present in a water solution of the target copolymer. In addition to high solvent polarity, the intermolecular and intramolecular electrostatic attraction between the indolenine cation and the COO^−^ anion were found to be influencing factors, which stabilize these MC form of spiropyran groups grafted onto CMCH. In a water solution, visible light bleaching was completed over a short period (8 minutes) under artificial visible light irradiation and the thermal coloration reaction, whose rate constant at 25 °C was 4.64 × 10^−4^ s^−1^, which fit the first-order reaction equation. After ten photochromic cycles in water solution, the relative absorption intensity of CMCH-g-MCA decreased by 7.92%.

## Introduction

**Scheme 1.**
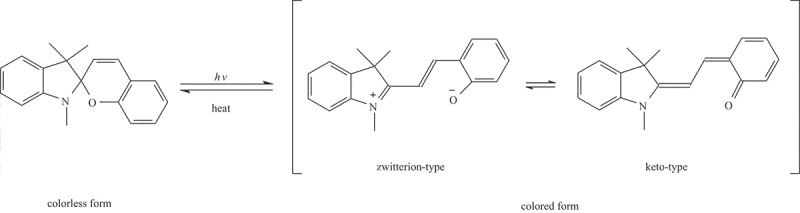
Photochromism diagram of spiropyran and two limiting structure of MC

The discovery of photochromic reactions of spiropyrans by Fischer and Hirshberg in 1952 and Hirshberg’s approach of applying the phenomenon to ‘photochemical erasable memory’ initiated active research on photochromism [1–3]. Typical examples of photochromic reactions of spiropyrans and the closely related spirooxazines are the reversible photochemical cleavage of the C–O bond in the spiro rings, which facilitates ring opening to give a merocyanine (MC) form (Scheme 1). Owing to their excellent properties, spiropyrans and spirooxazines have wide application prospects in various systems such as optical information storage devices [1–6], photochromic coatings [7,8], and molecular switches [9,10]. However, most of the MC forms of spiropyrans and spirooxazine are thermally unstable at room temperature, hindering their wide-scale commercial applications [11,12]. To overcome this limitation, these compounds are incorporated in polymer matrices, which can simplify device processing and improve the thermal fading stability of their MC forms owing to the steric-hindrance-induced retardation of chemical reactions [13–15]. Instead of using photochromic polymers doped with photochromic molecules, the application of photochromic polymers, wherein the photochromic groups are linked by covalent bonds, can overcome many limitations, such as phase separation of colorants.

Furthermore, most spiropyran and spirooxazine compounds are soluble in organic solvents and insoluble in water, which limits their applications. Thus, it is necessary to enhance the water solubility of these compounds. Their water-soluble derivatives can be prepared through introducing hydrophilic groups into their molecule structures [16–23], modifying water-soluble macromolecules with photochromic compounds [24–28], or copolymerizing photochromic vinyl compounds with water-soluble monomers [29–33].

Carboxymethyl chitin (CMCH) derivatives containing spirooxazine moieties have been prepared by Fu and coworkers [24]. The thermal fading stability is significantly enhanced, as expected, but the color of the product in a water solution gradually changes from blue to colorless when UV radiation is stopped. Thus, the closed form of spirooxazine groups grafted onto CMCH is more stable in water solutions than its MC form. This indicates that the MC form has a predominant keto-type structure in water because the zwitterion-type structure would be more stable in solvents with high polarity.

Although the MC forms of spirooxazines and most non-nitro-substituted spiropyrans have predominant keto-type structures, the MC forms of nitro-substituted spiropyrans particularly have a zwitterion-type structures [34,35]. Thus, grafting nitro-substituted spiropyrans onto water-soluble macromolecules may show different results. In this study, a novel water-soluble photochromic polymer was synthesized by graft copolymerization of the nitro-substituted spiropyran moiety onto CMCH, and its photochromic behavior in a water solution was investigated.

## Experimental section

### Materials and reagents

CMCH was supplied by Zhejiang Yuhuan Biochemical Co. Ltd (China). Its carboxymethylation substitutive degree was 0.65 and the average molecular weight was 3.0 × 10^5^ g/mol. It was purified before use by dissolution in deionized water and precipitation in acetone (two times), followed by extraction in a Soxhlet apparatus by refluxing in acetone for 24 h and drying at 60 °C under vacuum for 48 h. 1ʹ-(2-hydroxyethyl)-3,3ʹ-dimethyl-6-nitrospiro[2 *H*-1-benzopyran-2,2ʹ-indoline] (SP-OH, >93%, TCI); *N,N*-dimethylaminopyridine (DMAP, ≥99%, Sinopharm Chemical Reagent Co., Ltd); 1,3-dicyclohexylcarbodiimide (DCC, ≥99%, Sinopharm Chemical Reagent Co., Ltd); Dialysis bag (molecular weight cut-off of 8000–14,000 g/mol, Sinopharm Chemical Reagent Co., Ltd). All other chemicals were analytical reagents and used as supplied by the company.

### Synthesis of SPA monomer

**Scheme 2.**
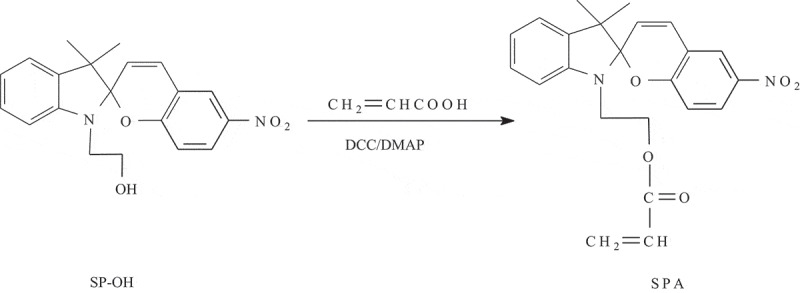
Synthetic route to SPA monomer

The synthetic route to the SPA monomer is outlined in Scheme 2. SP-OH (3.52 g), acrylic acid (0.79 g), DCC (2.07 g), DMAP (0.12 g), and anhydrous diethyl ether (50.0 mL) were added into a 100 mL round bottom flask. The mixture was stirred in the dark at 25 °C. The course of the reaction was monitored by thin layer chromatography. After completion of the reaction, the precipitate was removed by filtration. The filtrate was washed with 100 mL of 0.5 mol/L Na_2_CO_3_, 100 mL of saturated salt water, 100 mL of 0.5 mol/L HCl, and 100 mL of saturated salt water two times each and dried over anhydrous MgSO_4_ for 48 h. The final solution was evaporated under reduced pressure to produce the crude compound. The crude compound was recrystallized two times using ethyl acetate to give the product as a reddish solid (3.22 g) with a yield of 79.3%. m.p. 167–168 °C. IR (KBr) υ (cm^−1^): 3416, 3394, 3290, 2931, 2854, 1728, 1655, 1620, 1541, 1522, 1485, 1452, 1407, 1339, 1271, 1186, 1089, 955, 808, 746. ^1^ HNMR (CDCl_3_, 600 MHz) δ: 8.01(m,2 H), 7.22(m,1 H),7.10(d,1 H), 6.90(m,2 H), 6.75(d,1 H), 6.70(d,1 H), 6.38(dd,1 H), 6.06(dd,1 H), 5.87(d,1 H), 5.83(dd,1 H), 4.31(m,2 H), 3.54(t,1 H), 3.45(t,1H), 1.28(s,3 H), 1.16(s,3 H).

### Preparation and purification of CMCH-g-SPA

For the reaction, a 100 mL three neck round-bottomed flask with a stirrer in a temperature-controlled water-bath was used in the dark. A total of 0.40 g of CMCH was dissolved in 50.0 mL of deionized water, followed by the addition of 1.22 g SPA. After purging with purified nitrogen for 30 min, the mixture was stirred strongly and heated slowly to 70 °C and then 68.4 mg of ammonium persulfate in deionized water was added into the flask. The reactive mixture was stirred constantly and was allowed to react at 70 °C for 3 h. After cooling by water bath, the dialysis against distilled water (10 L × 3) was conducted (12 h × 3) to remove small water-soluble molecules [36–38]. After concentrating the solvent under reduced pressure, the residue was precipitated by pouring into excess acetone. The precipitate was filtered and extracted with acetone in a Soxhlet extractor for 24 h to remove the homopolymer. During the extraction, some acetone from the Soxhlet extractor was added dropwise to test paper. After exposure to UV light (254 nm), if the paper color remained the same it indicated that the homopolymer was completely removed. Subsequently, the purified CMCH-g-SPA was dried under vacuum at 60 °C for 48 h to give the product as a waxy, loose solid (0.684 g). The percentage of grafting (G%) was determined by the following equation (1):

(1) $$G\%\;=\;\left(w_{1}-w_{0}\right)/w_{0}\times 100\%$$

where *w_0_* and *w_1_* denote the weights of CMCH and CMCH-g-SPA, respectively.

### Characterization and property testing

The Fourier-transform infrared (FT-IR) spectra were recorded by a NEXUS-670 Fourier transform infrared spectrophotometer, and the samples were scanned from 4000 cm^−1^ to 400 cm^−1^ using KBr pallets.

The thermogravimetric (TG) analysis was performed by an SDT Q600 synchronous thermal analyzer under the nitrogen flow rate of 100 mL/min, and the samples were heated from room temperature to 650 °C at a heating rate of 10 °C/min.

X-ray diffraction (XRD) patterns were collected using a Japanese physics Rigaku D/Max-2400 powder X-ray diffractometer. The samples were scanned from 5° to 60° of 2θ in steps of 0.02°.

The UV-vis absorption spectra were determined by a UV-1800PC-DS2 spectrometer; the samples were scanned from 190 nm to 650 nm. A light emitting diode (flashlight of a mobile phone, 1 W) was used as the visible light source. The light emitting diode faced the center of the cuvette, and the distance between them was 3 mm.

### Evaluation and comparison of water solubility

The water solubility of CMCH and CMCH-g-SPA were evaluated as follows:

(a) The samples were ground to pass the 80 mesh sieve (aperture 180 μm);

(b) 0.06 g of samples was added to 10 mL deionized water under stirring for 24 h at 25 °C. Solubility performance was determined and compared by visual observation as to whether the mixture was uniform and whether there precipitated substances were present in the mixture.

(c) The above mixtures were poured on a large piece of glass. The glass was tilted to enable the mixture on the glass to flow down slowly. The resulting liquid membranes were spread out, so that the membrane was very thin. Solubility performance was determined and compared by visually observing whether the liquid membranes were uniform, whether the liquid membranes were transparent, and whether precipitated or insoluble substances were present.

(d) The liquid membranes on the glass were dried in the darkness under near vacuum at 30 °C. Solubility performance was determined and compared by observing whether the solid membranes were uniform, whether the solid membranes were transparent, and whether the solid membranes were smooth (by touching the thin solid membranes with hands).

(e) The solid membranes on the glass were completely peeled from the glass. Solubility performance was determined and compared by examining the toughness of the thin solid membranes by bending the solid membranes.

(f) If the results of the observations in steps b, c, d, and e were inconclusive, 0.02 g of the samples was added to perform multiple trials of steps b, c, d, and e.

### Photochromism kinetics

The visible light bleaching and thermal coloration of a 0.1 mg/mL water solution of the target copolymer were investigated. After the sample solution was prepared and placed in a dark room for 24 h, it was irradiated with visible light for specific time period, such as 60 s, 120 s, 240 s, 360 s, 480 s, 600 s; the absorption spectra were determined until constant absorbance was achieved. Then, the sample was placed in the darkroom for thermal coloration, and the absorption spectra were obtained at pre-designed time points, such as 2 min, 4 min, 6 min, 12 min, 24 min, 36 min, 1 h, 2 h, 3 h, 4 h, 5 h, 6 h, 7 h, 8 h, 9 h, 10 h, until constant absorbance was achieved. The reaction rate constants (*k*) of visible light bleaching and thermal coloration were separately calculated by the first-order reaction equation (2):

(2) $$-ln\left(A_{t}-A_{\infty }\right)\;=kt-\;ln\left(A_{0}-A_{\infty }\right)$$

where *A_0_, A_t_*, and *A_∞_* are the absorbance at the *λ_max_* at time zero, any time *t*, and time ∞, respectively. The half-life time (*t_1/2_*) was calculated by the following equation (3):

(3) $$t_{1/2}=\;0.693/k$$

### Fatigue resistance testing

The fatigue resistance of the target copolymer was tested at 25 °C. A 0.1 mg/mL water solution of the target copolymer was kept in the dark for 10 h and irradiated by visible light for 10 min. This was repeated for 10 cycles. For the n^th^ photochromic cycle, the absorbance at λ_max_ after keeping the sample in the dark for 10 h (A_open,n_) and the absorbance at λ_max_ immediately after 10 min of visible light irradiation (A_closed,n_) were determined and the relative absorbance change (η) of the n^th^ photochromic cycle was calculated by equation (4):

(4) $$\eta_{n}=\left[1-\left(A_{open,n}-A_{closed,n}\right)/\left(A_{open,1}-A_{closed,1}\right)\right]\times 100\%$$

## Results and discussion

### Copolymerization reaction

Through the functional group reaction, spiropyran or spirooxazine compounds with a specific functional group can be grafted onto natural macromolecules with functional groups they can react with. However, this approach has not been widely investigated because natural macromolecules have simple functional groups [25,26]. SPA (an acrylate of SP-OH) and methacrylate of SP-OH are common monomers having a vinyl group; thus, their copolymerization with other monomers [29–33,39–43] has been conducted to prepare and investigate many copolymers with interesting properties.

Chitin is one of the most abundant biopolymers identical to cellulose. Chitosan is the deacetylated product of chitin. Although their structures are similar, cellulose is a homopolymer, while chitin and chitosan are heteropolymers [44]. The presence of the amino and hydroxyl functional groups, which could easily be chemically modified, promotes the easy modification of chitin/chitosan [45]. As is well-known, CMCH is a chitin derivative with a sodium carboxymethyl group (-CH_2_COONa), which can replace either the hydroxyl or amino group.

**Scheme 3.**
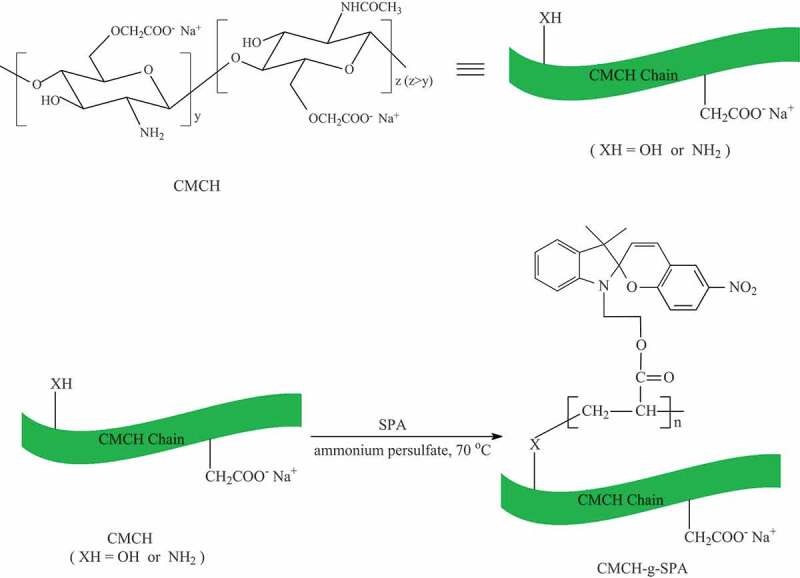
Synthetic route to CMCH-g-SPA

The graft polymerization of vinyl monomers onto carboxymethyl chitosan has gained significantly more attention than those onto CMCH because the amino functionality in carboxymethyl chitosan is greater than that in CMCH. Moreover, chitin has amino functionality, just less than that of chitosan [46]. Furthermore, the synthesis of carboxymethyl chitosan requires chitosan to first be prepared from chitin, which is followed by a carboxymethylation step; whereas, the CMCH synthesis requires only the carboxymethylation of chitin. In addition, the graft copolymerization of vinyl monomers with chitin/chitosan derivatives occurs not only because of amino functionality [47–49], but also because of hydroxyl functionality [47–51]. Thus, graft copolymerization of vinyl monomers with CMCH can be performed, which can be seen in Scheme 3[47–49].

Several CMCH-g-SPA with different G% were prepared by changing the reaction conditions. The G% of CMCH-g-SPA prepared according to the reaction conditions described in the experimental section was 71%, and this sample, along with CMCH, were characterized and the results are described below.

**Figure 1.**
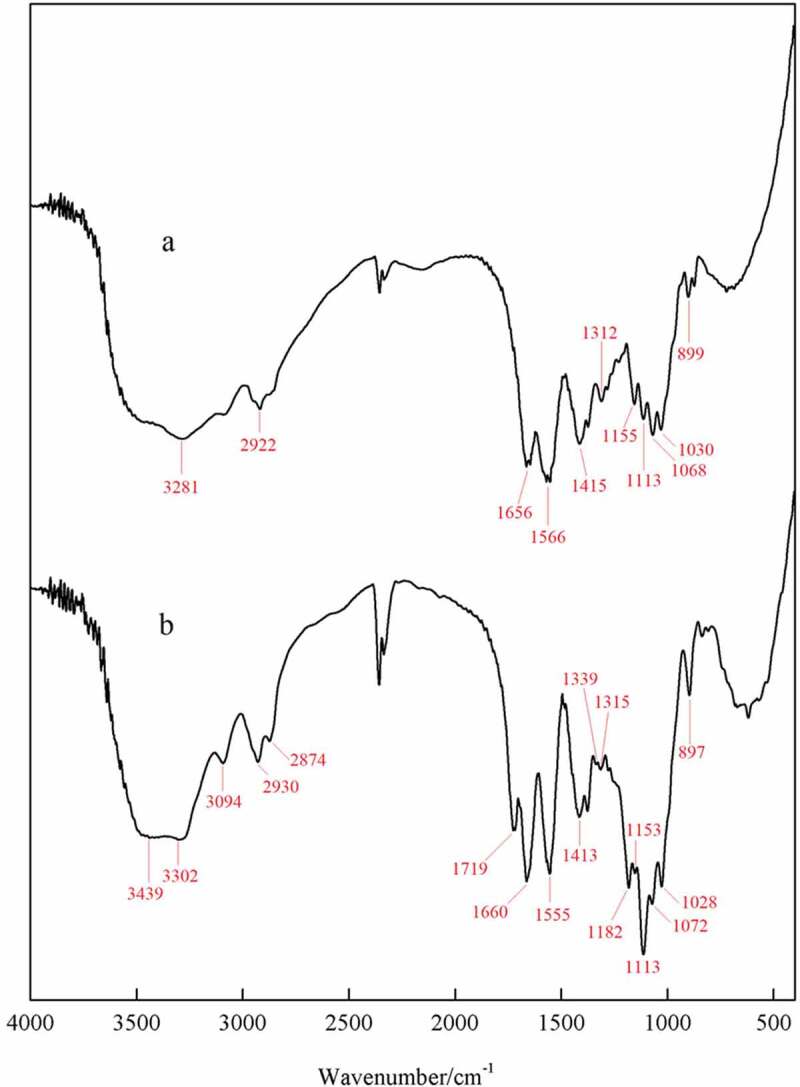
IR spectra of (a) CMCH and (b) CMCH-g-SPA

The IR spectra of CMCH and CMCH-g-SPA are shown in Figure 1. For CMCH, the band at 1656 cm^−1^ is assigned to the C = O stretching vibrations of the amide I [50–57]. This band overlaps with that arising from the asymmetric stretching of the carboxylate anion [58,59]. The band at 1566 cm^−1^ is assigned to the N–H stretching vibrations of the amide II [50,51,56], the band at 1415 cm^−1^ represents the symmetric stretching of the carboxylate anion, [53,56,57] and the band at 1312 cm^−1^ corresponds to the C–N stretching vibrations of the amide III. The band at 899 cm^−1^ is characteristic of the β-glucoside bond.

In the IR spectra of the CMCH-g-SPA, all characteristic bands of CMCH appear, but with different wavenumbers and intensity. The absorption band around 3270–3480 cm^−1,^ assigned to the stretching vibrations of the OH and NH groups, was narrow, indicating that grafting copolymerization occurred with either the free hydroxyl or the amino group. The broad absorption band around 1113 cm^−1^ that was assigned to the stretching vibration of the ether bond significantly increased, which is attributed to the addition of the aromatic ether in the pyran ring of spiropyran pendants (band at 1182 cm^−1^) and the formation of the ether bond by the vinyl monomers grafting onto the hydroxyl groups. By introducing numerous methyl and methylene groups, the intensity of their bands around 2874–2930 cm^−1^ increased significantly. Before grafting, the intensity of the band at 1656 cm^−1^ was lower than that of the band at 1566 cm^−1^. After grafting, this band moved to 1660 cm^−1^ and its intensity was greater than that of the band at 1555 cm^−1^. This change is because of the overlap of the bands assigned to the stretching vibrations of the C = C bond in the pyran ring of spiropyran pendants grafted onto CMCH.

**Figure 2.**
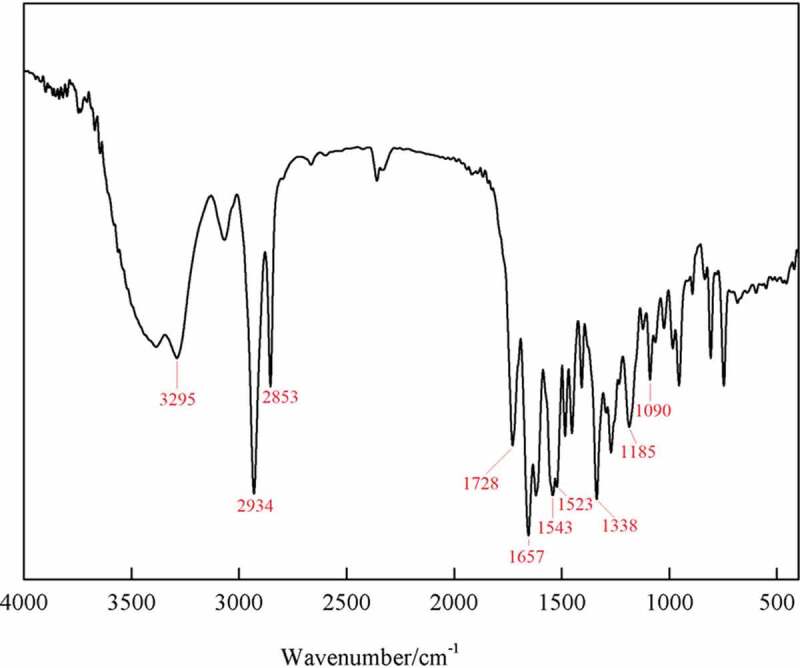
IR spectra of SPA homopolymer

In addition, some absorption bands not belonging to CMCH appeared, such as the characteristic absorption band of the stretching vibration of the C = O ester bond at 1719 cm^−1^; whereas, in the spectra of the SPA homopolymer (Figure 2), the band corresponding to the C = O stretching vibration appears at 1728 cm^−1^. The weak absorption band at 1339 cm^−1^ belongs to the symmetric vibrations of the nitro group, and the absorption band representing antisymmetric vibrations of the nitro group overlaps with the broad absorption band around 1555 cm^−1^. In contrast, in the spectra of the SPA homopolymer, the absorption bands for symmetric and antisymmetric vibrations of the nitro group appear at 1338 and 1523 cm^−1^, respectively. As the homopolymer was completely removed by acetone extraction, the presence of the above-mentioned bands in the spectra of CMCH-g-SPA confirms the occurrence of grafting.

**Figure 3.**
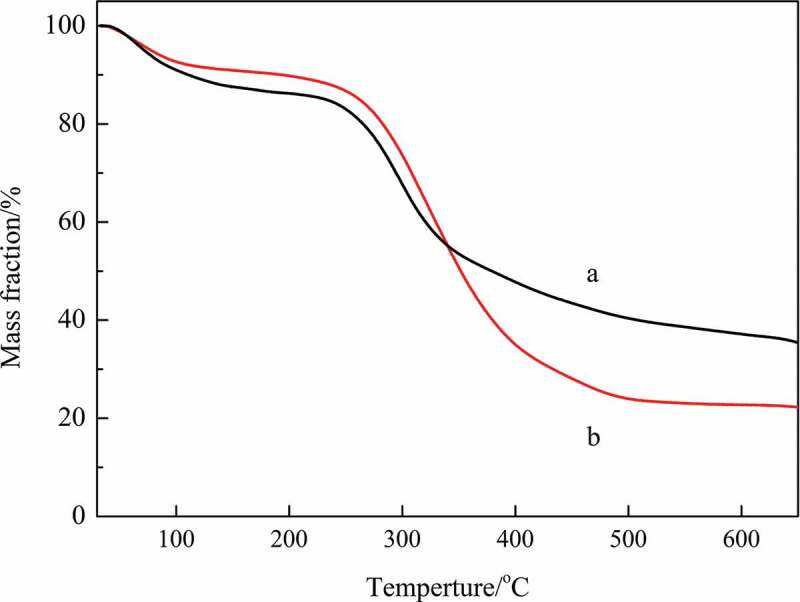
TG curves of (a) CMCH and (b) CMCH-g-SPA

The TG curves for the thermal degradation of CMCH and CMCH-g-SPA are shown in Figure 3. In the case of CMCH, three distinct weight-loss zones are observed from 30 °C to 650 °C. The initial, mild weight loss is due to evaporation of the remaining and bound water [53]. The second major weight loss occurred in the 240–350 °C temperature range with a mass loss of 30.28 wt%, which was due to the degradation of the CMCH structure [53]. The rate of weight loss is increased with increase of temperature. Then, a slow weight loss process emerges with a mass loss of 16.81 wt% from 350 °C to 600 °C. In the case of CMCH-g-SPA, two distinct weight-loss zones are observed. The first weight loss refers to evaporation of the remaining and bound water, but this weight loss is slightly smaller than that seen for CMCH. This phenomenon can be interpreted as the weakening of the ordered structure of CMCH by graft copolymerization, which was confirmed by XRD; thus, the amount of water trapped in the ordered structure was reduced. The second weight loss takes place in the 230–500 °C temperature range with a mass loss of 63.85 wt%. The beginning of this temperature range basically coincides with the previous, but its end is obviously delayed with a large mass loss. The wider temperature range and the greater mass loss can be attributed to the influence of the grafting copolymerization reaction. The weight loss slows down above 500 °C.

**Figure 4.**
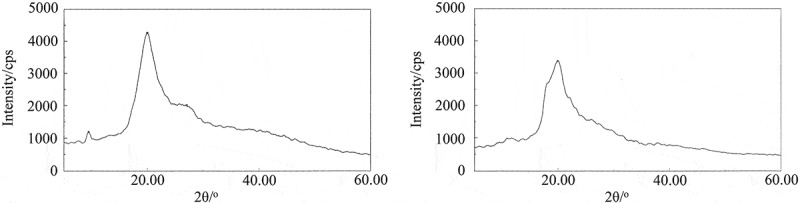
XRD patterns of CMCH (left) and CMCH-g-SPA (right)

On comparing of the XRD patterns of chitin [50,55,60–63] and CMCH [52–54,62,64], the position of XRD peaks shows little difference, but the intensity is markedly different. This implies that these XRD peaks originated from highly crystalline chitin with extensive hydrogen bonding that has been partly destroyed by carboxymethylation, resulting in the decrease in degree of crystallinity. The XRD patterns of CMCH and CMCH-g-SPA are shown in Figure 4. The diffraction pattern of CMCH shows two sharp peaks at 2θ = 9.48 and 2θ = 20.02, indicating the semicrystalline structure of CMCH [53,54]. For CMCH-g-SPA, the peak at 2θ = 9.48 vanishes, and the diffraction peak at 2θ = 19.90 shows a marked decrease; this implies that graft copolymerization further disrupts the CMCH semicrystalline structure resulting in the decrease in degree of crystallinity of CMCH-g-SPA [54,56,57,65].

Chitin is highly crystalline and insoluble in all common solvents [44,61,66]. The water-solubility of CMCH is not only due to the incorporation of carboxymethyl groups but also the destruction of its crystal structure by the carboxymethylation reaction [66]. SPA is soluble in organic solvent and insoluble in water. After grafted copolymerization, 0.12 g of CMCH-g-SPA can be dissolved in 10 mL water. CMCH-g-SPA has improved water-solubility compared to that of CMCH, and it is related to the further destruction of the crystal structure of CMCH by graft copolymerization.

Although chitin and chitosan have similar structures, spiropyran-functionalized-*N*-phthaloyl-chitosan is insoluble in water (assumed because the polymer is purified by water washing and its properties were investigated in an ether dispersion [67]), because the structures of the nitro-substituted spiropyrans used in these two experiments are similar. The chitin derivates prepared in this study show water-solubility, providing evidence that the water-soluble parent-CMCH attracts the spiropyran pendant groups into the water.

**Figure 5.**
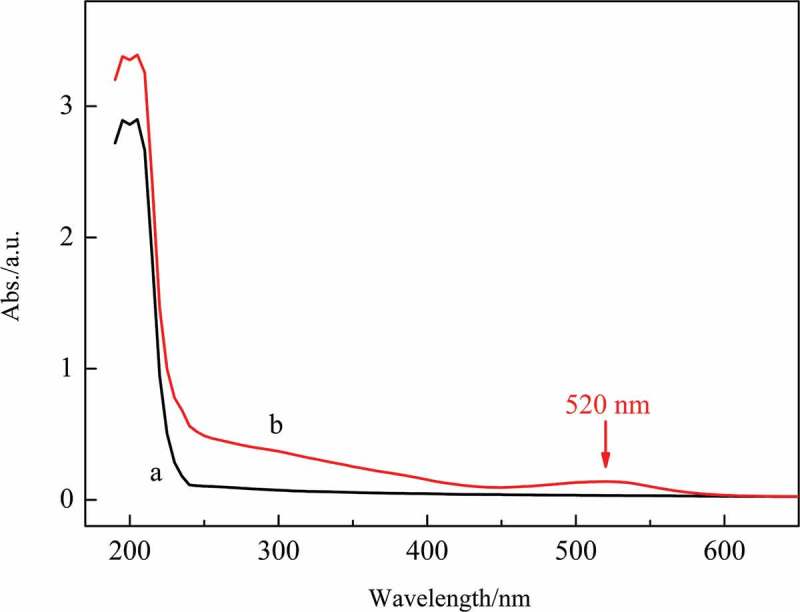
UV-vis spectra of (a) CMCH and (b) the target copolymer

The UV-vis spectra of CMCH and the target copolymer are shown in Figure 5. The spectrum of CMCH only shows strong absorption below 215 nm, which is assigned to n-π* electronic transitions of the carbonyl group in the COO^−^ anion and the amido group [68,69]. The UV-vis spectrum of the target copolymer showed absorption from 190 nm to 600 nm. Tyer reveals that the UV-vis absorption band of closed form spiropyran (Scheme 1) in a 3-methylpentane solution appears below 350 nm [70]. CMCH grafted copolymers without spirooxazine side groups do not absorb in the visible light region, and even with spirooxazine groups (before UV irradiation) they should not absorb in this region [24]; therefore, it was unexpected when our target copolymer showed an absorption band at 520 nm. Considering that the water solution of the target copolymer appears reddish, where greater solution concentrations give more intense color, and the wavelength of this absorption band is in the visible region [71], we conjectured that the MC form of spiropyran derivatives existed in the tested water solution and the origin of this absorption band in the visible region is from the MC form of spiropyran moiety grafted onto CMCH.

### Photochromic properties

**Figure 6.**
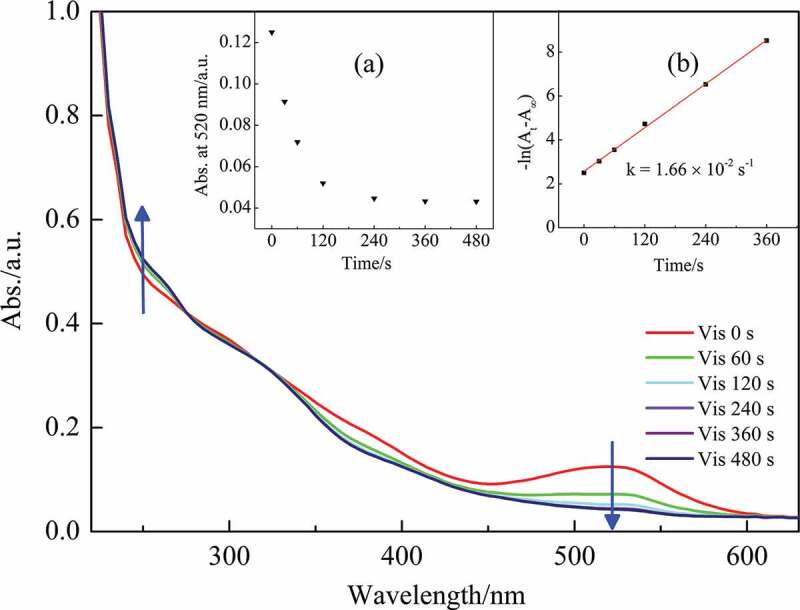
UV-vis absorption of CMCH-g-MCA during visible light bleaching (inset (a) shows absorption intensity changes at 520 nm as a function of irradiation time, and inset (b) shows the rate constant plot for the first-order reaction)

The presence of CMCH-g-MCA (MC form of CMCH-g-SPA) in water solution under the laboratory conditions without UV irradiation implies that CMCH-g-MCA has a better stability than that of the corresponding CMCH-g-SPA. After intensive visible light irradiation, the colored solution fades to colorless. The UV-vis spectra of a CMCH-g-MCA sample (0.1 mg/mL) in water irradiated by visible light over different times are shown in Figure 6. The absorption band intensity at 520 nm gradually decreased, meaning that the ring-closing reaction occurred, and colored CMCH-g-MCA translated into colorless CMCH-g-SPA under visible light irradiation.

Figure 6 inset (a) shows the intensity of absorption at 520 nm as a function of visible light irradiation times. This band decreased in intensity until 480 s of visible light irradiation had passed and the decrease then stopped, suggesting that the visible light bleaching attained a stationary state. The rate constant (*k*) for the visible light bleaching is 1.66 × 10^−2^ s^−1^ (see Figure 6 inset (b)). The *t_1/2_* is 41.75 s.

**Figure 7.**
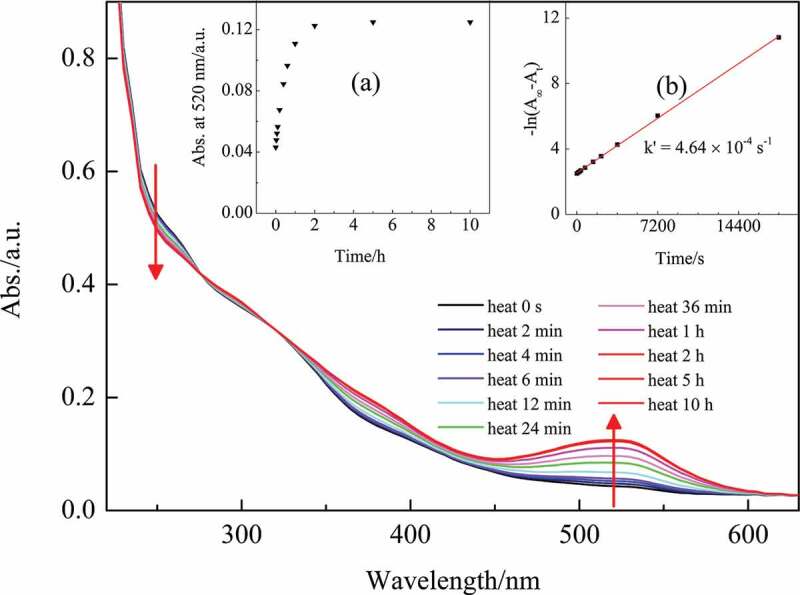
UV-vis absorption of CMCH-g-SPA during thermal coloration (inset (a) shows absorption intensity changes at 520 nm as a function of thermal coloration time, and inset (b) shows the rate constant plot for the first-order reaction)

This CMCH-g-SPA sample (after visible light bleaching was complete) was then placed in darkness for thermal coloration. The UV-vis spectra of this sample during the thermal coloration are shown in Figure 7. The absorption intensity of the band at 520 nm gradually increased with time, meaning that the ring-opening reaction occurred, and the colorless CMCH-g-SPA translated into the colored CMCH-g-MCA in the absence of visible light. Figure 7 inset (a) shows the 520 nm band absorption intensity as a function of thermal coloration time in the dark. After 10 h of thermal reaction, the increase in the 520 nm band almost stagnated, suggesting that thermal coloration attained a stationary state. The rate constant (*k*ʹ) for the thermal coloration was 4.64 × 10^−4^ s^−1^ (see Figure 7 inset (b)). The *tʹ_1/2_* was 24.89 min.

According to the definition of negative photochromism [72,73], CMCH-g-MCA is a negative photochromic dye. To the best of our knowledge, the negative photochromism of polymers linked covalently with spirooxazine groups has not been reported [72]. Because the MC form of spirooxazines has a predominant keto-type structure, polymers covalently linked to spirooxazine groups exhibit positive photochromic phenomena, even in water solutions [24], or when the polymers are polyelectrolytes. [74–76] In contrast with spirooxazines, the MC forms of some special spiropyran compounds, especially those with free nitro, carboxy, or sulfo groups, exhibit negative photochromic phenomena in a strong polar solvent [72,73]. This means that the MC forms of those spiropyran compounds essentially possess a zwitterion-type structure; therefore, the strong polar solvent stabilizes the MC.

Spiropyran compounds (nitro-substituted) supported by polyethylene glycol show positive photochromism in common organic solutions, but negative photochromism in water solutions [77]. A chitosan derivative modified by nitro-substituted spiropyrans, spiropyran-functionalized-*N*-phthaloyl-chitosan, shows positive photochromic phenomena in ether dispersions [67]. In contrast, chitin derivates prepared in this work show negative photochromism in water solutions. This indicates that the polarity of the solvent influences the stability of MC.

The waterborne polyurethane derivative containing nitro-substituted spiropyran groups shows positive photochromic phenomena in water dispersions [78]. The spiropyran compounds (nitro-substituted) supported by polyethylene glycol show negative photochromism in water solutions [77]. This may be attributed to the fact that it is difficult for the dispersed substance to have full contact with water in dispersions; thus, the MC groups in water dispersions are not stabilized by water. Besides polarity of the solvent, the properties of polymer main chains or copolymers also affect the stability of MC.

The enzyme-bound spiropyran exhibited normal photochromism when the matrix enzyme was hydrophobic in nature; whereas, negative photochromism was evident when the spiropyran was attached to hydrophilic enzymes [25]. These phenomena indicate that the photoisomerization of enzyme-bound spiropyran is affected by the characteristics of the polymer main chain.

The polymer prepared by copolymerization of 1,3,3-trimethyl-6-methacryloyloxyspiro(2 *H*-1-benzopyran-2,2′-indoline) (MSP; 3.0 mol%) with 2-(dimethylamino)ethylmethacrylate (DMAEMA) exhibits negative photochromism in a polar solvent [33]. The formation of the colored MC before UV irradiation was applied was attributed to the stabilization of the zwitterion-type character of the MC in the polar media induced by the polar DMAEMA comonomer units. Under the same conditions, polymers prepared by the copolymerization of MSP and methyl methacrylate exhibited positive photochromism, which was attributed to the nonpolar character of the MMA comonomer, which does not stabilize the MC. These phenomena indicate that the stability of MC is affected by the characteristics of copolymers.

**Figure 8.**
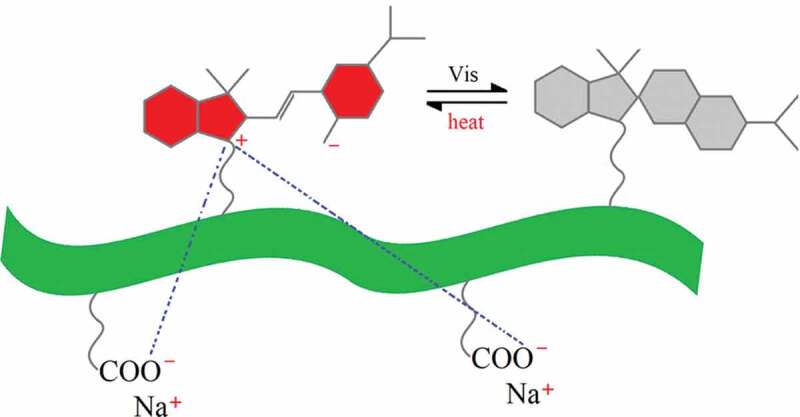
Illustration of intermolecular and intramolecular electrostatic attraction of the target copolymer

With the above analysis, it can be concluded that the negative photochromic phenomenon of CMCH-g-MCA is due to not only the structure of MC and the polarity of the solvent, but also the properties of the CMCH main chain. CMC is a type of water-soluble anionic linear polymer. The zwitterion-type character of MC has two different charge centers: the indolenine cation and the phenoxy anion. As a well-known fact, opposite charges attract each other [75,76]; thus, it is predicted that the electrostatic forces are developed not only between the indolenine cation and the phenoxy anion, but also between the indolenine cation and the COO^−^ anion (Figure 8). The intermolecular and intramolecular electrostatic attraction between the indolenine cation and the COO^−^ anion is another considerably influencing factor that can stabilize the MC moieties grafted onto the CMCH main chain.

### Fatigue resistance

Another limiting factor regarding the industrial application of spiropyran/spirooxazine photochromic compounds is the fatigue phenomenon after long-term use. Spiropyran derivatives exhibit worse fatigue resistance than spirooxazine derivatives [79–82]. The fatigue resistance of spiropyran/spirooxazine derivatives can be improved by adding appropriate antioxidants or hindered amine light stabilizers or UV stabilizers [83,84]; introducing antioxidant group into spiropyran/spirooxazine molecule structure [84,85]; or other methods [86].

**Figure 9.**
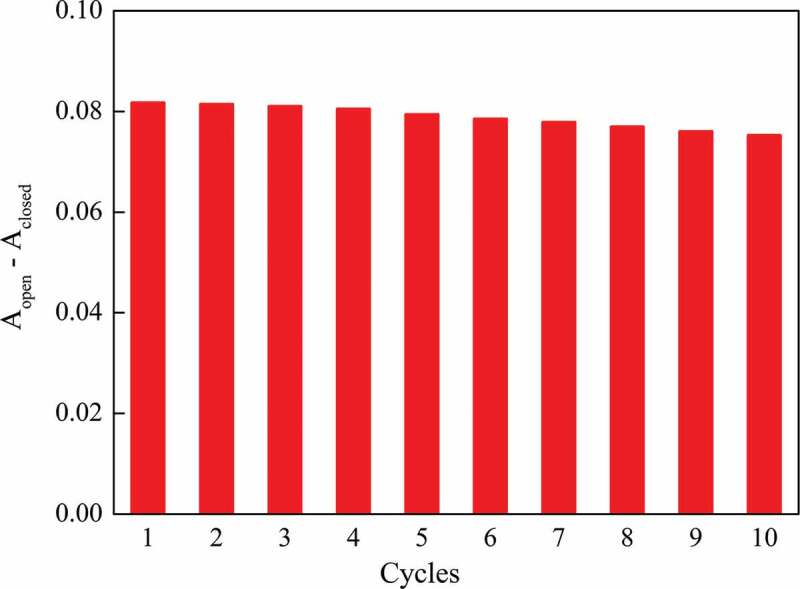
Relative absorbance during photochromic cycles at 25 °C

One of the ways to investigate the fatigue resistance of spiropyran/spirooxazine derivatives is to monitor the relative absorbance change (η) at λ_max_ in every photochromic cycle [32,78,87,88]. As shown in Figure 9, ten photochromic cycles of the target copolymer were conducted, and a maximum decrease of 7.92% in relative absorbance was observed. Compared with the waterborne polyurethane containing spiropyran groups (same evaluation method, ten photochromic cycles) [78], the target copolymer exhibited better fatigue resistance.

Chitin [89–94] and some of its derivatives (including carboxylated derivatives [94–96], grafted copolymer [97], and others) exhibit antioxidant activity and serve as scavenging free radicals. The better fatigue resistance of the target copolymer may be attributed to its structure, especially, to the original repeating units of chitin that had not been chemically modified previously.

## Conclusions

A nitro-substituted spiropyran acrylate, 1ʹ-(2-acryloxyethyl)-3,3ʹ-dimethyl-6-nitrospiro[2 *H*-1-benzopyran-2,2ʹ-indoline], was synthesized and grafted onto water-soluble carboxymethyl chitin macromolecule to obtain a water-soluble photochromic copolymer. The structure of the target copolymer was characterized by FT-IR spectroscopy, TG analysis, XRD, water-solubility tests, and UV-vis spectrophotometry. Its XRD pattern implies that graft copolymerization disrupts the carboxymethyl chitin semicrystalline structure, resulting in the improvement of water-solubility characteristics of the target copolymer. The target copolymer in the water solution appears reddish under natural conditions, and after exposure to visible light the copolymer turns colorless. On placing the solution into darkness, it reverts to its reddish appearance. The negative photochromic behavior of the target copolymer in water solutions was investigated and the cause was analyzed. In addition to the strong polarity of the solvent, the intermolecular and intramolecular electrostatic attraction between the indolenine cation and COO^−^ anion also considerably influence the stabilization of the open form of the target copolymer. After ten photochromic cycles in water solution, the target copolymer shows relatively good reversible photochromic behavior, this probably because that chitin exhibit antioxidant activity and serve as scavenging free radicals.
